# Résection-anastomose de la trachée cervicale sur écrasement trachéal post-traumatique

**DOI:** 10.11604/pamj.2015.22.268.8277

**Published:** 2015-11-20

**Authors:** Rex Mario Razafindrakoto, Herimalalaniaina Angelo Valisoa

**Affiliations:** 1 Service d'Oto-rhino-laryngologie et de Chirurgie Cervico-faciale, Centre Hospitalier Universitaire d'Andohatapenaka, Antananarivo, Madagascar

**Keywords:** Résection-anastomose, tomodensitométrie, trachée, traumatisme, Sleeve resection, CT scan, trachea, trauma

## Image en medicine

Un patient, de genre masculin, âgé de 30 ans, a présenté un traumatisme cervical antérieur par choc direct sur le volant de sa voiture au cours d'un accident de la voie publique. Le bilan clinique lésionnel au service des urgences chirurgicales a retrouvé un patient dyspnéique avec des reliefs cervicaux antérieurs peu modifiés, sans lésions cutanées objectivables. Un examen naso-laryngo-fibroscopique a permis de noter un rétrécissement de la lumière trachéale en infra-glottique avec des plis vocaux mobiles (A). Un examen tomodensitométrique cervical sans injection de produit de contraste en coupe coronale a montré une sténose trachéale à hauteur de la septième vertèbre cervicale (B), et une fracture avec écrasement d'anneaux trachéaux en coupe axiale (C). Une exploration chirurgicale cervicale sous anesthésie générale a été réalisée une semaine après l'accident. Une résection-anastomose trachéale a été faite, portant sur cinq anneaux et sur une hauteur de deux centimètres et demie. Le rapprochement des parties proximale et distale de la trachée saine a d'abord été effectué au niveau de la partie postérieure et musculaire de la trachée (D), et ensuite sur ses faces latérales et sur sa face antérieure. Une canule de trachéotomie à ballonnet a été mise en place en fin d'intervention (E). Le cou du malade a été maintenu en hyperflexion par des sutures mento-sternales (E) laissées en place pendant deux semaines jusqu'à complète cicatrisation et jusqu'au retour au domicile, sur un patient décanulé et eupnéique.

**Figure 1:**
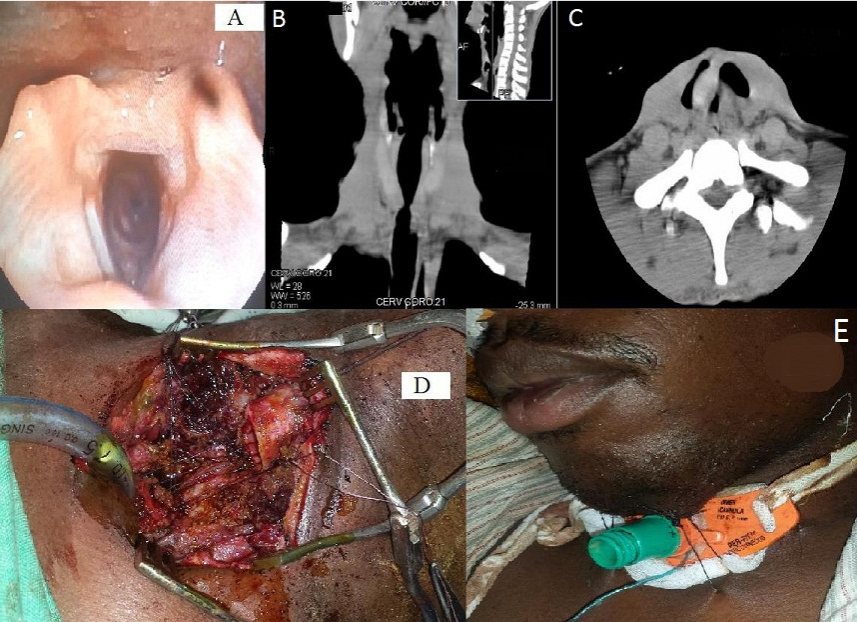
A: vue endoscopique en naso-laryngo-fibroscopie montrant la sténose infra-glottique et trachéale; B: tomodensitométrie cervicale en coupe coronale montrant une sténose trachéale; C: tomodensitométrie en coupe axiale montrant une fracture et un écrasement trachéaux; D: suture trachéale; E: fixation du cou en hyperflexion en fin d'intervention

